# Prevalence of Insomnia and Its Associated Factors in the General Population of Saudi Arabia: A Cross-Sectional Study

**DOI:** 10.7759/cureus.44342

**Published:** 2023-08-29

**Authors:** Ahmed Metwally, Abdulbari D Alalawi, Ali A Al Sarrar, Osamah M Alamin, Ahmed A Saad, Meshari D Almalki

**Affiliations:** 1 Family and Community Medicine, Taibah University, Madinah, SAU; 2 College of Medicine, Taibah University, Madinah, SAU

**Keywords:** sleep condition indicator, dsm-5, associated factors, risk factors, saudi arabia, general population, prevalence, insomnia

## Abstract

Introduction: Insomnia is a common sleep disorder that can have negative impacts on daily functioning and health. However, little is known about the prevalence of insomnia and its associated factors in the general population of Saudi Arabia. This cross-sectional study aimed to investigate the prevalence of insomnia and its associated factors among adults in Saudi Arabia.

Methods: A self-administered electronic questionnaire was distributed to 4818 participants from all 13 regions of Saudi Arabia. The eight-item Sleep Condition Indicator (SCI) based on the Diagnostic and Statistical Manual of Mental Disorders, Fifth Edition (DSM-5) criteria was used to assess the prevalence of insomnia.

Results: The results showed that 37.6% of participants met all the DSM-5 clinical criteria for insomnia in the past month. The prevalence was higher among females, divorced or widowed individuals, students, unemployed individuals, those living with friends or family, those reporting severe work stress, and those using mobile devices before sleeping. On the other hand, the prevalence was lower among males, married individuals, morning and evening employees, those who practice physical exercise, and those with good health status.

Conclusion: This study found the prevalence of insomnia to be 37.6% in the general population of Saudi Arabia, which is considerably high. The risk factors associated with insomnia in the Saudi population were found to be age, sex, work stress, and using mobile devices before sleeping, while protective factors included being employed, practicing physical exercise, and having good health status. Further research is needed to explore the impact of insomnia on the quality of life and productivity of individuals in Saudi Arabia.

## Introduction

Insomnia is a sleep disorder that is marked by having problems initiating or maintaining sleep, or early-morning awakening with an inability to return to sleep, even with adequate opportunities and conditions for sleep, and producing significant consequences in the form of distress or impairment in daily functioning [1,2]. It can present independently or as a symptom of other medical or psychological conditions. Insomnia can be divided into chronic and short term. Chronic insomnia is when the previous symptoms persist for at least three months, with a frequency of at least three times weekly. Short-term insomnia is when the symptoms persist for one to three months and are usually triggered by a major life stressor. Significant consequences on daily functioning can happen due to a lack of energy, low mood, or focusing problems [1,2].

It is important to take insomnia seriously, as it has a negative impact on both the country’s economy and the patient’s health [3]. Shortage of sleep is associated with productivity loss, road traffic accidents, and increased economic drain [4]. Also, insomnia can lead to an increase in comorbidities, as it is associated with an increased risk of hypertension, diabetes, cardiovascular diseases, dementia, and psychological disorders [5-9].

Insomnia is a common sleep disorder. Estimating the global prevalence of insomnia can be challenging, due to the use of different definitions and instruments. For instance, a study found that the weighted prevalence of insomnia based on the Diagnostic and Statistical Manual of Mental Disorders (DSM)-IV and DSM-5 criteria in the same population group is 22.1% and 10.8%, respectively, presenting a difference of more than 50% [10]. Another study measured the prevalence of short-term insomnia on 66 sites around 13 countries and found that 11.3% of the 57,298 participants were suffering from insomnia. They also found that insomnia was associated with female gender, age above 30 years old, religious practice, alcohol, and drug use disorders [11].

Among Middle Eastern countries, studies on the prevalence of insomnia in the general populations are lacking. Two studies have estimated the prevalence of insomnia in the general population of Turkey. One was published in 2014, and the other one in 2016; they found the prevalence to be 12.2% and 15.3%, respectively [12,13]. In Lebanon, one study estimated the prevalence to be as high as 47.1% [14].

Most of the studies conducted among the Gulf Cooperation Council (GCC) countries were directed at specific populations from college students and healthcare providers to primary care patients. Researchers from Kuwait University tried to estimate the prevalence of insomnia among the general population of the GCC countries using the Arabic version of the Insomnia Severity Index (ISI). During the COVID-19 lockdown, they distributed an online questionnaire through social media. They found the prevalence of insomnia to be 66.7%, 63.9%, 64.4%, 48.4%, 61.4%, and 63% in UAE, Kuwait, Saudi Arabia, Oman, Qatar, and Bahrain, respectively. With a total of 63.9% across all six countries, which is high compared to the rest of the world [15]. In Bahrain, a study estimated the prevalence to be 17.4% using the ISI [16]. In Qatar, it was estimated to be 3.0% using the DSM-5 diagnostic criteria or 5.5% using the Sleep Condition Indicator (SCI) cut-off score to define insomnia [17].

In Saudi Arabia, about 50% of the population sleeps less than seven hours a day [18]. Although this is a significant number of people suffering from lack of sleep, the information available regarding the prevalence of insomnia in our general population is limited, as most of the studies are directed at specific population groups. One of the few studies that have attempted to estimate the prevalence of insomnia in the general population of Saudi Arabia was performed on a sample of 2095 healthy participants visiting King Abdulaziz Medical City in Riyadh, Saudi Arabia. The prevalence of insomnia was 77.7% [19]. Two studies were conducted on the prevalence of insomnia in the Saudi primary care population. The first study was done in Riyadh. The prevalence was found to be 76.4%, and when daytime consequences were included, it decreased to 57.1% [20]. The second study was in Aseer, and the prevalence was found to be 60.1% [21]. This high prevalence in the primary care population could be a significant problem, due to the lack of knowledge on sleep medicine and its disorders by primary care physicians in Saudi Arabia [22].

A study was done on Saudi college students during the COVID-19 pandemic, which found that 52.6% of Saudi undergraduate students had sleep disruption, which had a significant association with anxiety, depression, and stress [23]. A similar study showed that insomnia prevalence in Saudi college students during the COVID-19 pandemic was 41% [24].

Our study aims to estimate the prevalence of insomnia in the general population of Saudi Arabia and to determine the factors associated with it. Knowing the prevalence of insomnia will help in assessing the extent and the impact of the problem in the country. Identifying the risk factors and possible protective factors of insomnia among the Saudi population can help in decreasing the prevalence of insomnia in our society.

## Materials and methods

Study design

This is a quantitative, cross-sectional, observational study. An internet-based self-administered questionnaire was distributed to the general population in the 13 regions of Saudi Arabia. It was distributed from June 11, 2022, to July 6, 2022. We included participants who were above the age of 18 years and lived in Saudi Arabia. We excluded individuals who refused to participate in the study, individuals under 18 years old, or individuals who cannot read Arabic or English.

Sample size

The study was conducted in 13 regions of Saudi Arabia. The adult population of Saudi Arabia is 26 million. The number of participants necessary to ensure a confidence level of 95% in the population of Saudi Arabia was calculated by Raosoft (Raosoft, Inc., Seattle, WA) using an estimated insomnia prevalence of 18% and a 3% error margin. An estimate of 385 samples was suggested. A total of 4818 participants completed the questionnaire.

Sampling method and measurement tool

A self-administered anonymous electronic questionnaire created on Google Forms (Google, Mountain View, CA), both in English and Arabic, was used to assess the prevalence of insomnia and associated factors among adults in Saudi Arabia using convenience sampling. It was distributed using data collectors from different regions in Saudi Arabia. The questionnaire was made up of four sections. The first section was a consent form to be included in the research. The second section asked for socio-demographic information. The third section included the eight-item SCI based on the DSM-5 criteria. Each item was scored on a five-point Likert scale (0-4). The scale items measure sleep continuity, sleep satisfaction, symptoms severity, personal functioning, daytime performance, and duration of sleep disturbance. The score of all items ranged from 0 to 32, with a lower score indicating sleep disturbance. We used the validated cut-off score of 16 or less to represent the percentage of our sample that met the minimum criteria for assumed insomnia disorder [25]. The translated Arabic version of the scale was validated and applied to a similar population of Saudi Arabia [17]. The fourth section asked about factors that might be associated with insomnia.

Ethical considerations

Ethical approval (Study ID: STU-21-007) was obtained from Taibah University, College of Medicine Research Ethics Committee (CM-REC) on January 30, 2022. The study was compiled according to the Declaration of Helsinki. Participant-identifying data were not collected. The objective of the research was explained to the participants. They had the right to take part or refuse to participate in the study. Participants were informed that their information would be confidential and would be used for research purposes only. We did not offer incentives or rewards to the participants.

Statistical analysis

The collected data were entered and analyzed using the Statistical Package for Social Sciences version 22.0 (IBM Corp., Armonk, NY). The characteristics of the studied subjects were tabulated and presented in frequency (number and percentage). The prevalence of DSM-5 clinical criteria in the past month among the studied subjects was calculated and its 95% confidence interval (CI) was estimated. A comparison of DSM-5 insomnia with the studied subjects’ characteristics was done using the chi-square test. P-value ≤ 0.05 was used as a level of statistical significance. Univariate logistic regression analysis was used to calculate the odds ratio (OR) and its 95% CI for the association of insomnia with the studied subjects' factors. A predictive stepwise regression model was used to investigate the most important factors associated with insomnia, where all significant associated factors in the univariate analysis were included in the stepwise model, with a p-value of 0.05 as the entry criterion and a p-value of 0.10 as the exclusion criterion.

## Results

**Figure 1:**
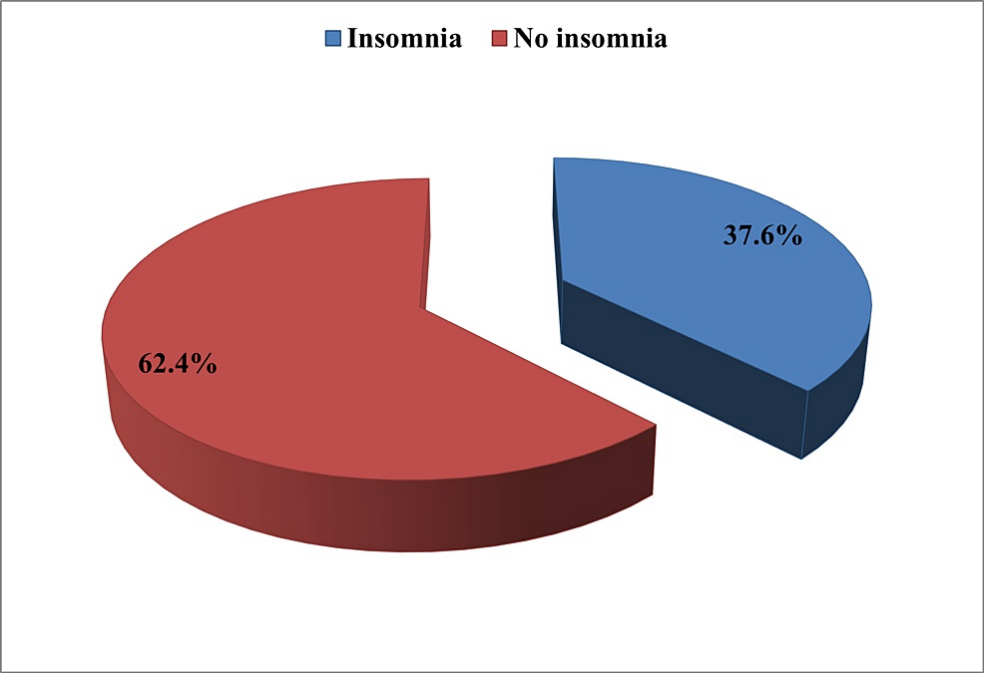
Prevalence of DSM-5 insomnia disorder among the studied sample Prevalence of DSM-5 clinical criteria for insomnia in the past month in the studied 4818 subjects. DSM-5: Diagnostic and Statistical Manual of Mental Disorders, Fifth Edition.

The study analyzed data from 4818 out of 4863 (99%) participants who completed and returned the study questionnaire. Figure 1 shows that the prevalence of subjects who met all the DSM-5 clinical criteria for insomnia in the past month was 37.6% (n = 1813; 95% CI = 36.2-39.1%).

Table 1 presents the socio-demographic personal characteristics of the studied subjects where subjects aged < 35 years represented 64.8% of the sample and 59.6% were females. About three-fourths of the sample were university and higher educated, half were single, and one-fourth were unemployed. The majority of the studied subjects reported living with their families (91.7%), being non-smokers (82.6%), and using mobile before sleeping (90.5%). More than half of the sample reported moderate work stress (59.4%), intake of caffeinated drinks every day (56.3%), and good health status (50.2%).

**Table 1:** Socio-demographic and personal characteristics of the studied population DSM-5: Diagnostic and Statistical Manual of Mental Disorders, Fifth Edition.

Characteristics	Total participants (n = 4818)
	Number (percentage)
Age in years	
<35	3120 (64.8%)
35-<50	1057 (21.9%)
50-65	588 (12.2%)
>65	53 (1.1%)
Sex	
Male	1948 (40.4%)
Female	2870 (59.6%)
Education	
Less than secondary	243 (5.0%)
Secondary	935 (19.4%)
University and higher	3640 (75.6%)
Marital status	
Single	2475 (51.4%)
Married	2124 (44.1%)
Divorced or widowed	219 (4.5%)
Job	
Unemployed	1245 (25.8%)
Employed (morning shift)	1961 (40.7%)
Employed (evening shift)	302 (6.3%)
Student	1310 (27.2%)
Living	
Alone	271 (5.6%)
With friends	131 (2.7%)
With family	441 (91.7%)
Smoking	
No	3978 (82.6%)
Yes	840 (17.4%)
Stress at work	
No	921 (19.1%)
Moderate	282 (59.4%)
Severe	1034 (21.5%)
Caffeinated drinks	
No	333 (6.9%)
1-5/week	1774 (36.8%)
Every day	2711 (56.3%)
Physical exercise	
No	3040 (63.1%)
Yes	1778 (36.9%)
Mobile use before sleep	
No	460 (9.5%)
Yes	4356 (90.5%)
Health rating	
Poor	164 (3.4%)
Fair	1050 (21.8%)
Good	2417 (50.2%)
Excellent	1187 (24.6%)
DSM-5 insomnia	
No	3005 (62.4%)
Yes	1813 (37.6%)

Table 2 presents the distribution of DSM-5 clinical criteria for insomnia in the past month by the studied subjects' characteristics. The prevalence of DSM-5 insomnia showed significant variations in the studied subjects’ factors. The prevalence has a statistically significant relation among subjects aged 35-50 years (39.9%), females (41.2%), divorced and widow (43.4%), students (41.7%), unemployed (41.1)%, living with friends (41.2%), those reported severe work stress (63.5%), not practice physical exercise (43%), intake caffeinated drinks every day (40.3%), use mobile before sleeping (38.8%), and among those with poor (74.4%) and fair (57.6%) health rating.

**Table 2:** Distribution of DSM-5 clinical criteria for insomnia in the past month by the studied subjects' characteristics * Significant; DSM-5: Diagnostic and Statistical Manual of Mental Disorders, Fifth Edition.

Characteristics	Insomnia (n = 1813)	No insomnia (n = 3005)	P-value
	Number (percentage)	Number (percentage)	
Age in years			
<35	1179 (37.8%)	1941 (62.2%)	0.03*
35-<50	422 (39.9%)	635 (60.1%)	
50-65	197 (33.5%)	391 (66.5%)	
>65	15 (28.3%)	38 (69.2%)	
Sex			
Male	630 (32.3%)	1318 (67.7%)	<0.0001*
Female	1183 (41.2%)	1687 (58.8%)	
Education			
Less than secondary	81 (33.3%)	162 (66.7%)	0.35
Secondary	350 (37.4%)	585 (62.6%)	
University and higher	1382 (38.0%)	2258 (62.0%)	
Marital status			
Single	970 (39.2%)	1505 (60.1%)	0.004*
Married	748 (35.2%)	1376 (64.8%)	
Divorced and widow	95 (43.4%)	124 (56.6%)	
Job			
Unemployment	512 (41.1%)	733 (59.9%)	<0.0001*
Employment (morning shift)	662 (33.8%)	1299 (66.2%)	
Employment (evening shift)	93 (30.8%)	209 (69.2%)	
Students	546 (41.7%)	74 (58.3%)	
Living			
Alone	66 (24.4%)	205 (75.6%)	<0.0001*
With friends	54 (41.2%)	77 (58.8%)	
With family	193 (38.3%)	2723 (61.7%)	
Smoking			
No	1511 (38.0%)	2467 (62.0%)	0.27
Yes	302 (36.0%)	538 (64.0%)	
Physical exercise			
No	1307 (43.0%)	1733 (57.0%)	<0.0001*
Yes	506 (28.5%)	1272 (71.5%)	
Stress at work			
No	125 (13.6%)	896 (86.4%)	<0.0001*
Moderate	1031 (36.0%)	1832 (64.0%)	
Severe	657 (63.5%)	377 (36.5%)	
Caffeinated drinks			
No	122 (36.6%)	211 (63.4%)	<0.0001*
1-5/week	599 (33.8%)	1175 (66.2%)	
Every day	1092 (40.3%)	1619 (59.7%)	
Mobile use before sleep			
No	123 (26.7%)	337 (73.3%)	<0.0001*
Yes	1690 (38.8%)	2668 (61.2%)	
Health rating			
Poor	122 (74.4%)	42 (25.3%)	<0.0001*
Fair	605 (57.6%)	445 (42.4%)	
Good	856 (35.4%)	151 (64.6%)	
Excellent	230 (19.4%)	957 (80.6%)	

Table 3 shows the association of DSM-5 insomnia with the studied subjects' factors. A statistically significant positive association was found between DSM-5 insomnia and some studied factors where the risk was high among subjects reported living with friends (OR = 2.20; 95% CI = 1.39-3.40) and with their families (OR = 1.95; 95% CI = 1.45-2.57), those reported moderate (OR = 3.60; 95% CI = 2.92-4.39) and high (OR = 11.1; 95% CI = 8.85-13.9) work stress, and those using mobile before sleeping (OR = 1.75; 95% CI = 1.40-2.15). A significant negative association, however, was found between DSM-5 insomnia and subjects' sex, marital status, job, and health status. The risk of insomnia was significantly reduced among males (OR = 0.68), married subjects (OR = 0.84), morning employees (OR = 0.73), evening employees (OR = 0.63), and those who reported physical exercise practice (OR = 0.52). The risk was markedly reduced by 54%, 82%, and 92% among subjects with fair, good, and excellent health ratings with OR of 0.46, 0.18, and 0.08, respectively.

**Table 3:** Univariate logistic regression analysis for the association of DSM-5 clinical criteria for insomnia in the past month with the studied subjects' factors * Significant; OR: odds ratio; CI: confidence interval; Ref.: reference; DSM-5: Diagnostic and Statistical Manual of Mental Disorders, Fifth Edition.

Factors	Insomnia (n = 1813)	No insomnia (n = 3005)	OR	95% CI
Age in years				
<35	1179	1941	1	Ref.
35-<50	422	635	1.1	0.96-1.27
50-65	197	391	0.83	0.68-0.99*
>65	15	38	0.65	0.36-1.19
Sex				
Male	630	1318	0.68	0.60-0.77*
Female	1183	1687	1	Ref.
Education				
Less than secondary	81	162	1	Ref.
Secondary	350	585	1.2	0.88-1.60
University and higher	1382	2258	1.22	0.93-1.61
Marital status				
Single	970	1505	1	Ref.
Married	748	1376	0.84	0.75-0.95*
Divorced and widow	95	124	1.2	0.90-1.57
Job				
Unemployment	512	733	1	Ref.
Employment (morning shift)	662	1299	0.73	0.63-0.85*
Employment (evening shift)	93	209	0.63	0.48-0.83*
Students	546	74	1.02	0.87-1.20
Living				
Alone	66	205	1	Ref.
With friends	54	77	2.2	1.39-3.40*
With family	193	2723	1.95	1.45-2.57*
Smoking				
No	1511	2467	1	Ref.
Yes	302	538	0.92	0.78-1.07
Physical exercise				
No	1307	1733	1	Ref.
Yes	506	1272	0.52	0.46-0.60*
Stress at work				
No	125	896	1	Ref.
Moderate	1031	1832	3.6	2.92-4.39*
Severe	657	377	11.1	8.85-13.9*
Caffeinated drinks				
No	122	211	1	Ref.
1-5/week	599	1175	0.88	0.68-1.12
Every day	1092	1619	1.17	0.92-1.46
Mobile use before sleep				
No	123	337	1	Ref.
Yes	1690	2668	1.75	1.40-2.15*
Health rating				
Poor	122	42	1	Ref.
Fair	605	445	0.46	0.32-0.67*
Good	856	151	0.18	0.13-0.27*
Excellent	230	957	0.08	0.05-0.12*

Table 4 displays the predictors of DSM-5 insomnia among the studied subjects. The most important factors increasing the risk of insomnia among the studied subjects were age, work stress, and mobile use before sleeping. The risk was significantly higher among those with moderate (OR = 2.75), and severe stress at work (OR = 7.35) and those who reported using mobile before sleeping (OR = 1.60). On the other hand, however, the risk was significantly reduced among employed subjects, those who practice physical exercise, and students where the estimated OR was 0.65, 0.55, 0.70, and 0.75, respectively. Fair, good, and excellent health status of the studied subjects were associated with a marked significant reduction of the risk of insomnia with estimated OR of 0.54, 0.27, and 0.17, respectively.

**Table 4:** Predictors of DSM-5 insomnia among the studied subjects: the result of stepwise predictive regression model * Significant; OR: odds ratio; CI: confidence interval; Ref.: reference; DSM-5: Diagnostic and Statistical Manual of Mental Disorders, Fifth Edition.

Characteristics	Insomnia (n = 1813)	No insomnia (n = 3005)	OR	95% CI
Age in years				
<35	1179	1941	1	Ref.
35-<50	422	635	1.2	1.01-1.40*
50-65	197	391	0.9	0.70-1.12
>65	15	38	0.8	0.40-1.60
Job				
Unemployment	512	733	1	Ref.
Employment (morning shift)	662	1299	0.65	0.56-0.79*
Employment (evening shift)	93	209	0.55	0.40-0.73*
Students	546	74	0.75	0.63-0.93*
Stress at work				
No	125	896	1	Ref.
Moderate	1031	1832	2.75	2.22-3.40*
Severe	657	377	7.35	5.77-9.34*
Health rating				
Poor	122	42	1	Ref.
Fair	605	445	0.54	0.36-0.80*
Good	856	151	0.27	0.17-0.39*
Excellent	230	957	0.17	0.10-0.22*
Physical exercise				
No	1307	1733	1	Ref.
Yes	506	1272	0.7	0.62-0.81*
Mobile use before sleep				
No	123	337	1	Ref.
Yes	1690	2668	1.6	1.23-2.10*

## Discussion

In this cross-sectional study, we aimed to estimate the prevalence of insomnia and identify the factors that might be associated with it in the general population of Saudi Arabia. We found that 37.6% of the participants in our sample had insomnia. Also, we found that being a woman, being between the ages of 35 and 50 years, being divorced or widowed, being unemployed, and living with friends were all related to a higher risk of insomnia. Other factors linked to an increased risk of insomnia were having moderate or high levels of work stress, using a phone right before bed, and having poor health status. On the other hand, the study found that a lower prevalence of insomnia was associated with being male, being married, engaging in physical activity, and having a good or exceptional health status.

Compared to the previous studies done in Saudi Arabia, we found a significantly lower percentage of the prevalence of insomnia, which is probably due to the use of different tools and different populations. The other studies estimated the prevalence of insomnia in the general population of Saudi Arabia to be 77.7% using an instrument based on the International Classification of Sleep Disorders, 2nd edition (ICSD-2), in the Saudi primary care population to be 76.4% using the Pittsburgh Sleep Quality Index (PSQI), and in the Aseer primary care population to be 60.1% using the Athens Insomnia Scale (AIS) [19-21]. Another study was done among GCC countries to study the impact of COVID-19 and estimated the percentage of insomnia in the general population of Saudi Arabia to be 64.4% using ISI [15].

Overall, our study offers insightful information about the prevalence and risk factors for insomnia. The research findings can help medical practitioners and legislature to create effective prevention and management plans for insomnia.

To the best of our knowledge, this is the largest sample size for insomnia research in Saudi Arabia to date. Also, we used a tool that is based on the DSM-5 and was validated in both Arabic and English.

Our study has limitations that should be mentioned. Firstly, we had low male participation and excluded participants under the age of 18 years, which may not be representative of the population. Secondly, we used convenient sampling to acquire our sample, which can lead to selection bias. Thirdly, we used a self-administered anonymous electronic questionnaire, which may lead to response bias. Fourthly, we did not investigate the impact of important mental and medical conditions on insomnia due to the concern of classification bias in self-administered electronic surveys. Future research should use a representative sampling method and collect longitudinal data with potential multiple diagnosis criteria to address these limitations.

## Conclusions

This study found the prevalence of insomnia to be 37.6% in the general population of Saudi Arabia, which is considerably high. The risk factors associated with insomnia in the Saudi population were found to be age, sex, work stress, and using mobile devices before sleeping, while protective factors included being employed, practicing physical exercise, and having good health status. These findings highlight the need for interventions and awareness campaigns to address the risk factors associated with insomnia and promote protective factors to reduce the prevalence of insomnia in the Saudi population. Further research is needed to explore the impact of insomnia on the quality of life and productivity of individuals in Saudi Arabia.
